# Identifying medical students at risk of underperformance from significant stressors

**DOI:** 10.1186/s12909-016-0565-9

**Published:** 2016-02-02

**Authors:** Tim J. Wilkinson, Jan M. McKenzie, Anthony N. Ali, Joy Rudland, Frances A. Carter, Caroline J. Bell

**Affiliations:** University of Otago, Christchurch, P O Box 4345, Christchurch, 8140 New Zealand

## Abstract

**Background:**

Stress is associated with poorer academic performance but identifying vulnerable students is less clear. A series of earthquakes and disrupted learning environments created an opportunity to explore the relationships among stress, student factors, support and academic performance within a medical course.

**Methods:**

The outcomes were deviations from expected performances on end of year written and clinical examinations. The predictors were questionnaire-based measures of connectedness/support, impact of the earthquakes, safety, depression, anxiety, stress, resilience and personality.

**Results:**

The response rate was 77 %. Poorer than expected performance on all examinations was associated with greater disruptions to living arrangements and fewer years in the country; on the written examination with not having a place to study; and on the clinical examination with relationship status, not having the support of others, less extroversion, and feeling less safe. There was a suggestion of a beneficial association with some markers of stress.

**Conclusion:**

We show that academic performance is assisted by students having a secure physical and emotional base. The students who are most vulnerable are those with fewer social networks, and those who are recent immigrants.

## Background

Part of a good educational programme includes supporting students. The need for support differs across the student body – some will need more support than others. High distress levels among tertiary students are common, not just in medical students [1] and are negatively associated with academic performance [2–6]. There is a curvilinear relationship between stress and performance on complex tasks – either too little or too much stress can result in underperformance whereas there is a point where just enough stress is optimal [7]. Identifying students who are stressed or in need of support is not always straightforward. The direction of causality between stress and academic performance can also be difficult to unravel – is the association because stress causes the poor performance or because poor performance causes stress?

The 2010-2011 earthquakes in Christchurch, New Zealand, were stressful for students and staff. It did, however create a unique opportunity to explore the relationships among stress, student factors, support and academic performance. It also created some serendipitous methodological advantages: firstly it helped unravel the problem of direction of causality as the stressors were at the same time for all the students – this meant we could survey levels of stress in the students after some of the events, yet before the measures of academic performance (end of year exams) had occurred. Secondly, the primary stressor was universal – that is it affected all the students in the class, albeit not in identical ways. In relation to a curvilinear association between stress and performance, this meant the stimulus was controlled for, so the focus can be on the individuals’ responses. Finally, and as described later, we had a previously developed model that predicts performance on examinations, and thereby a mechanism to control for prior ability of students. These are strengths of the study in our view. Whereas many studies of stress look at how varying levels of stress affect performance, the factors above combined to make it possible to look at factors that make students vulnerable to stress.

### Conceptual framework

We draw on the work of Lazarus [8] and Tomaka [9], as applied to health professional education by Le Blanc [10]. A person’s response to a stressful situation is influenced by their assessment of that situation: initially they assess the demands required to continue to reach the required goal and then they assess the resources available to meet that demand (personal and/or environmental). If their resources are judged to be sufficient, then the situation is regarded as a challenge. If the resources are judged to be insufficient, then the situation is regarded as a threat. This means a situation regarded as stressful by one individual, may not be perceived as stressful for another [11, 12]. The perception of the stress is also dependent on the relationship between the stressor and the task, and factors such as coping styles, locus of control, and social supports [10]. Elevated stress levels can impede performance on tasks that require divided attention, working memory, retrieval of information from memory, and decision making [10].

Events viewed as being a challenge tend to lead to positive responses (studying harder, for example), while those viewed as being a threat tend to lead to negative responses, such as avoidance or dropping out [12, 13]. Furthermore, if the stressor is related to the task, there can sometimes be a beneficial effect. In contrast, if the stressor is unrelated to the task, performance is more likely to be impaired [10]. This may be explained by stress related to a task drawing more attention to that task, thereby creating the potential for enhanced performance on that task. In contrast, stress unrelated to a task will draw attention away from that task and onto the source of the stress instead [10]. Divided attention tasks, those that require the integration of information from several sources, are more vulnerable to the effects of stress [10].

Individuals who have access to psychological support when under stress seem to be in better health compared with individuals without significant support [10, 14]. Stress can be moderated by learned resourcefulness where a person is able to regulate internal events such as emotions and cognitions [2]. Coping styles can be determined by personality but they are also modified according to the social context [11, 14].

Students studying in a different country to that of their birth can experience different stressors, and sometimes more pronounced stress [15]. International medical students have been shown to be more at risk than domestic students with respect to test anxiety [16]. Those with less robust social support are more at risk of stress after a natural disaster [17, 18]. Stress can also be more pronounced if overseas students lack the necessary language skills to enable them to study competently in a language other than their own [12]. They also have less access to family, social and community support networks.

### This study

There has been a call for more research into this area, including identifying how a university can best become aware of students undergoing a crisis or stress and discovering the best interventions to assist these students in the short and long terms [5]. It is also important to understand better the contributions of various factors to performance under stress and to effectively prepare trainees to perform under acutely stressful conditions [10] and to enhance resilience [19]. Thus, while this study is set within the context of earthquakes, the findings could have relevance to other crises faced by institutions: not just natural disasters, but acts of terrorism, bomb threats or other stressors that affect groups of students.

The findings of this study are able to help determine the interactions among stress, student factors and examination performance when exposed to an institutional and community crisis. We have previously shown that unexpected disruptions to a learning environment can impair performance but, if there is time to adapt, students can often compensate [20]. Despite this, we hypothesized that there would be subsets within the student group who are less able to adapt. The aim of this study is to explore associations with poorer than expected academic performance following major stressful events, in order to identify those groups of medical students who may be at greatest risk.

### The context of the study

**Fig. 1:**
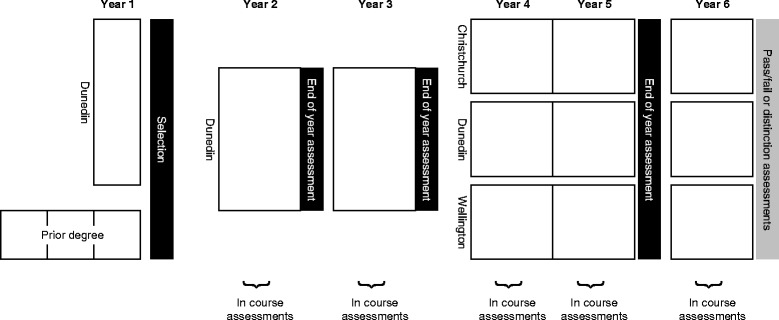
Outline of University of Otago medical course and timing of summative assessments

The Christchurch campus is part of the University of Otago medical school in New Zealand. The Otago medical course runs over 6 years and is represented schematically in Fig. 1. Admission into the course occurs into year 2 and follows either a prior degree or a common first year health sciences course. Years 2 and 3 are common to all students and are delivered in Dunedin. For years 4-6 the students study and live in Dunedin, Christchurch or Wellington. Students have end of year assessments (in October/November) in years 2, 3 and 5 that are common to each class year. The year 2 and 3 assessments are all undertaken in Dunedin while the year 5 assessments are identical but undertaken in the three different cities. There are two major components to the end of year 5 assessments: a written exam consisting of 4 h of multiple choice questions and 2 h short answer questions; and a 10 station practical examination in the form of an Objective Structured Clinical Examination (OSCE). The end of year exams commence in late October each year.

We have shown, across several cohorts, that students who perform well in the end of year 5 assessments have often performed well in the earlier years with an r range of 0.52-0.79 [20]. It is therefore possible to use the results from year 2 and 3 assessments to develop a predictive model for performance on end of year 5 assessments.

In 2010 and 2011 Christchurch, New Zealand, was struck by a series of powerful earthquakes. The students included in this study experienced a magnitude 7.1 earthquake in September 2010 near the end of their 4^th^ year of study, a magnitude 6.3 aftershock earthquake in February 2011 near the beginning of their 5^th^ year of study, and a further series of major aftershocks throughout the year. The magnitude 6.3 aftershock earthquake, although smaller in magnitude, was of shallow depth and close to the central city, thereby resulting in the highest ever recorded ground accelerations in a major city. There was widespread damage to the city and infrastructure. The health system was placed under considerable stress as 6659 people were injured and 182 died in the initial 24 h. During the earthquake the city hospitals were subjected to severe shaking, where staff could not stand unaided. Electricity supply was intermittent. 440 aftershocks rocked the city in the initial 24 h. The local emergency ambulance service had to evacuate its communication centre [21]. However, the hospital buildings remained usable throughout.

The main medical school building suffered damage resulting in it being unsafe to enter until it had been repaired. Repairs, and the need for further strengthening, necessitated the building’s closure for the 2011 and 2012 academic years and therefore loss of access to tutorial rooms, lecture theatres, student common rooms, laboratory space and the library. The clinical simulation centre was also closed for most of 2011. The houses of many staff members and students were damaged. A building that housed the departments of General Practice and Public Health was severely damaged and had to be demolished. Fortunately there was no loss of life of staff members or students. Students attended information sessions on the likely psychological effects and on how to access support.

The medical course could not be delivered at all for two weeks but the course then partially resumed. The hospital buildings, and most general practices, could still be used so most clinical experiences could continue. The medical school was able to obtain the use of a local golf club to deliver large group lectures. Teaching staff however were distracted by the health needs of their patients and the disruptions to their personal and professional lives.

In addition to the 2 major earthquakes described above, a further magnitude 6.3 earthquake struck the city in June 2011. Between September 2010 and the end of the 2011 academic year there were over 10,000 aftershocks, that impacted on both students and staff. A number of these aftershocks contributed to further damage in the city.

## Methods

Ninety 5^th^ year medical students from the University of Otago Christchurch campus were emailed inviting them to participate in an electronic survey asking them about their experiences relating to the earthquakes.

Surveys were sent at the beginning of September 2011, which was approximately two months prior to their end of year exams, and 3-12 months after the major earthquakes. If students did not respond, three email reminders were sent over the course of the next month. Students were given relevant information about the survey at the outset, and consented to participate in the survey. The study was approved by the University of Otago Ethics Committee.

### Data collection and analysis

#### Examination results

The outcome measures for this study were deviations from the predicted performances on the end of year 5 written examination and practical examination, and its combined mark (weighted 60 % for the practical examination and 40 % for the written examination).

In order to account for differences in student ability we used a previously developed multivariate regression model for students in the same class who were living in different cities to predict year 5 performance based on previous year 2 and year 3 performance. This predictive model satisfied the conditions for a linear regression, has been published elsewhere and has been shown to be stable across several cohorts of students [20]. We then determined score residuals by calculating mean differences (and 95 % confidence intervals) between the predicted results and actual results. A positive residual meant a student performed better than expected, based on their prior performance. In order for a reader to interpret effect sizes, the residuals represent absolute examination percentage score differences. For example, a residual of +1 means a student scored 1 mark higher out of 100 marks than expected; a residual of -1.5 means a student scored 1.5 marks lower out of 100 marks than expected.

#### Questionnaire survey

The survey [22] was designed to assess a broad range of domains to enable us to evaluate the impact of the earthquakes on students’ functioning. Within each domain we chose the best available instruments, while attending to brevity to avoid question overload. The particular domains of interest were the degree of stress experienced, elements of support and social connectedness that might protect against the effect of stress, and co-existing premorbid psychiatric conditions that might mediate any effect of stress. The questionnaire took 20-30 min to complete. A description of the range of responses has been published previously [22].

### Connectedness/support

Students reported how many years they had spent living in New Zealand and their relationship status (single, in a relationship, or married/de facto/civil union). Students rated (on 5-point scales) how supported they felt by the university and by people in their life in general (1 = not at all; 5 = extremely).

### Impact on living/personal space

Students rated the severity of impact of the earthquakes on their life using the following variables: living arrangements, having a place to study at home, and the library service. Severity of impact was rated on 4-point scales (1 = none; 4 = severe).

### Psychological scales and safety

#### Depression, Anxiety and Stress Scale (DASS)

The DASS measures self-rated current (past week) symptoms of depression, anxiety and stress [23]. The present study used the 21 item version of the scale, which produces comparable results to the longer version [24, 25]. The DASS yields a total score indicating overall severity of symptomatology (all domains combined), plus subscale totals for depression, anxiety and stress. Subscale totals are categorized as follows: normal, mild, moderate, severe and extreme. To ease interpretation, these categories were dichotomized as follows: normal-mild (≤13) and moderate-extreme (≥14).

#### Post-Traumatic Stress Disorder Checklist –Specific Event (PCL-S)

The PCL-S assesses self-rated current (past month) symptoms of post-traumatic stress disorder in relation to an identified stressful experience [26]. The scale consists of 17 items. A score of ≥44 is taken as indicating the presence of PTSD. In order to differentiate between general traumatic events and the earthquakes, the respondents were asked to respond to the survey “in the last 2 months in relation to the earthquake or aftershock that was the worst for you”.

#### Connor Davidson resilience scale

The Connor Davidson Resilience Scale assesses self-rated current (past month) resilience [27]. The scale consists of 25 items.

#### Eysenck personality questionnaire (Brief version)

The Eysenck Personality Questionnaire (Brief Version) assesses self-rated personality characteristics amongst adults [28]. The scale consists of 24 items. In the present study, students were asked to retrospectively rate their characteristics prior to the earthquakes. The associations between introversion /extraversion and arousal [29] and social support [30] justified inclusion of this measure.

#### Work and adjustment scale

The Work and Adjustment Scale assesses current self-rated impairment attributable to an identified problem, in this case earthquakes and aftershocks [31]. Five items assess work, home management, social leisure activities, private leisure activities and family and relationships.

#### Perception of Safety

Students rated the impact of the earthquakes on how safe they currently felt living in Christchurch (1 = very safe; 5 = not at all safe).

#### Alcohol use

Students rated the severity of impact of the earthquakes on alcohol use (1 = none; 4 = severe).

### Data analysis

Three multivariate linear regression models were developed, one for each combination of the three assessment outcomes.

Pearson correlation coefficients were used to compare correlations between the questionnaire variables (predictors) and the examination result residuals (outcomes). Student’s *t*-test, or analysis of variance, was used to compare examination results when scale results could be classified into 2 or 3 categories. The normality of the residuals was confirmed by visual inspection of the residual plots, which confirmed the appropriateness of these parametric analyses.

The primary aim of this observational study was to explore multiple univariate factors which might predispose individuals to risks associated with stressors and thereby inform future studies or interventions. As such there are no corrections for multiple comparisons. There is therefore a possibility of Type I errors associated with some of the conclusions.

The analyses were undertaken on anonymised data and complied with the requirements of the University of Otago ethics committee.

## Results

### Response rates

The response rate was 77 % (69/90). For some subscales, not all students completed sufficient questions and the actual numbers of respondents for each scale are given in the tables. Demographic details of the sampled population have been published previously [22].

Table 1 outlines correlations between the measures of connectedness and support, and the examination residuals. This shows that students who have lived in New Zealand for fewer years had worse than expected performance on their examinations. Those students who were not married or in a relationship also performed worse than expected, particularly in the practical examination. Although not reaching statistical significance, there were consistent negative associations between levels of support and exam performance – that is, students who felt unsupported by the university did better than expected. In contrast, those who felt supported by people in their life, in general, performed significantly better.

**Table 1:** Connectedness/support

		Exam residual - total	Exam residual - written	Exam residual - practical
How long have you lived in NZ (years) (n = 69)	r	.573	.411	.578
	p	<0.001	<0.001	<0.001
Relationship status				
Single	Mean	-1.864	-1.506	-2.103
In a relationship	Mean	1.075	0.402	1.523
Married/defacto/civil union	Mean	1.950	0.286	3.060
	F	3.705	1.043	4.922
	p	0.03	0.358	0.01
Overall, how supported have you felt by your university (n = 67)	r	-0.225	-0.189	-0.227
	p	.066	.125	.065
Overall, how supported have you felt by people in your life in general (n = 68)	r	0.267	0.117	0.251
	p	.028	.340	.039

Table 2 shows an adverse effect of disrupted living arrangements on expected examination performance. In particular, disruption to study space and perceived disruption to the library was associated with worse than expected performance on the written examination, while disruption to living space was associated with worse than expected performance on the clinical examination.

**Table 2:** Living/personal space

		Exam residual - total	Exam residual - written	Exam residual - practical
What has been the impact of the earthquakes on your living arrangements - currently (n = 68)	r	-.376	-.242	-.399
	p	.002	.047	.001
How much have the loss or disruption to the following affected your study?				
Having a place to study at home - currently (n = 66)	r	-.218	-.296	-.129
	p	.078	.016	.300
Changes to the library service - currently (n = 67)	r	-.163	-.230	-.090
	p	.188	.061	.467

Table 3 outlines correlations between the psychological and safety measures and examination residuals. This shows that people scoring higher on the PTSD checklist performed better than expected on the written examination and on the combined examination results. People who scored highly in extroversion performed better than predicted on the practical examination. People who felt less safe living in Christchurch performed worse than expected on the practical examination in comparison with those from other regions not directly affected by the earthquakes. People who felt the earthquakes had a greater impact on alcohol use performed better than expected on all examination results. There were notable lack of associations with exam performance and measures of resilience, anxiety, stress and depression. Those 8 students who scored ≥14 on the DASS depression subscale tended to have lower than predicted examination scores compared with those who scored in the normal range, but these differences were not statistically significant. Likewise, there were no significant differences in predicted examination scores for those scoring within the abnormal range for the other DASS subscales, or on the measure of PTSD.

**Table 3:** Psychological scales and safety

		Exam residual - total	Exam residual - written	Exam residual - practical
DASS depression (n = 64)	r	-0.088	0.044	-0.149
	p	0.491	0.728	0.241
Normal-mild; ≤13 (n = 56)	Mean	0.013	-0.181	0.142
Moderate-extreme; ≥14 (n = 8)	Mean	-0.879	0.238	-1.623
	t	0.511	0.232	0.857
	p	0.610	0.817	0.395
DASS anxiety (n = 63)	r	-0.066	0.014	-0.103
	p	0.608	0.914	0.423
Normal-mild; ≤9 (n = 57)	Mean	-0.436	-0.622	-0.312
Moderate-extreme; ≥10 (n = 6)	Mean	0.855	1.434	0.470
	t	0.631	0.945	0.327
	p	0.531	0.349	0.745
DASS stress (n = 65)	r	0.108	0.158	0.058
	p	0.392	0.209	0.646
Normal-mild; ≤18 (n = 56)	Mean	-0.347	-0.590	-0.186
Moderate-extreme; ≥19 (n = 9)	Mean	0.358	1.141	-0.163
	t	0.415	-.959	0.011
	p	0.679	0.341	0.991
Post-traumatic stress checklist (n = 64)	r	.259	0.225	0.234
	p	0.038	0.073	0.063
Normal; <44 (n = 53)	Mean	-0.519	-0.655	-0.428
PTSD; ≥44 (n = 11)	Mean	0.743	0.863	0.663
	t	-0.805	-0.907	0.597
	p	0.424	0.368	0.553
Eysenck Personality Questionnaire Extroversion (n = 61)	r	.318	0.176	.347
	p	0.013	0.174	0.006
Eysenck Personality Questionnaire neuroticism (n = 62)	r	0.169	0.191	0.124
	p	0.188	0.137	0.335
Connor Davidson Resilience Scale (n = 57)	r	0.021	-0.095	0.090
	p	0.878	0.484	0.504
Work and Social Adjustment scale (n = 63)	r	0.095	0.071	0.091
	p	0.457	0.577	0.473
How safe feel living in Christchurch (n = 66)	r	-.195	-.015	-.294
	p	.111	.903	.015
Impact of earthquakes on alcohol use (n = 64)	r	.301	.239	.288
	p	.014	.053	.019

## Discussion

Following a major stressful event affecting a learning environment, the students who were the most vulnerable, as measured by examination performance, were those who reported a greater disruption to their living arrangements and those who had spent fewer years in the country. These students were the most vulnerable, regardless of how they were examined (written, practical, or overall). For the written examination, there was an additional association with not having a place to study. For the practical examination, there were additional associations with relationship status, having the support of others, extroversion, and feeling less safe.

There were notable lack of associations between exam performance and measures of course delivery, resilience, anxiety and depression. The lack of associations with measures of psychological morbidity reflects the student group coming from a healthy, non-clinical, population and there being a low number of people with adverse scores.

Perhaps surprisingly, students who indicated that they had some specific difficulties or complaints, performed either no worse or *better* on some examinations. For example, those who felt less supported by the university, who reported a greater impact on alcohol use and who scored higher on measures of posttraumatic stress performed no worse on examinations. One interpretation of these findings is that these measures identified students who were more uneasy with the difficult situation they were in, and that these attributes had some beneficial impact in terms of performance on some examinations, possibly by prompting them to work harder. The DASS stress scale was not statistically significant, but the direction of the association is consistent with this hypothesis.

There were some effects that were dependent on the examination format. The association with living arrangements, particularly between study space at home and the written examination, is not surprising – studying for examinations requires time and space. The trend of an impact of disrupted library space on study and its effect on examination performance also supports this conclusion.

The effect on the practical examination was more complex, where feelings of safety, a student’s relationship status (marital status, support from people in their life), and markers of extroversion impacted on performance. Our interpretation of this is that the practical examination (an OSCE) is a more complex task, made up of more components and therefore needs more things to be going right for a student to perform. This interpretation is supported by noting that divided attention tasks, those that require the integration of information from several sources, are more vulnerable to the effects of stress [10]. The practical examination may therefore be more “sensitive” to disruptions, particularly support from others – which may attenuate the effect of feeling unsafe, but is synergistic with recent immigration. We believe these are novel observations. As well as being helpful in clinical interactions in general, extroversion may help in creating these support networks.

Being in a position to help others has been associated with lower levels of stress [32]. Recent immigrants may have less robust social networks – this could impact not just on their ability to receive support but also on their ability to provide help to others. Those with less robust social support are more at risk of stress after a natural disaster [17, 18]. Furthermore, an Australian study found that only 45 % of international medical students had their own GP and were more likely to feel uncomfortable accessing help outside the university [33]. We suggest two other possible mediating effects: community cohesion from a shared experience, and inclusion within a community of practice. The stressors experienced in this cohort were unusual in being universal, that is all the students shared the same stressors. Raphael describes a honeymoon phase deriving from the altruistic and ‘therapeutic community’ response in the period immediately following a disaster [34]. This can create a sense of community and cohesion where all are ‘pulling together’. This, in turn, could result in more collaborative learning preparation for examinations. The second mediating effect relates to inclusion within a community of practice [35]. It is possible the impact on the health service could contribute to the students’ motivation to become health professionals and their contributions during the aftermath could give them a greater sense of legitimate participation. However, we are also aware of anecdotes where the students also felt less included because of the attention being diverted to the injured. These areas would benefit from more in depth qualitative enquiry.

There are some important limitations in this study. It is cross-sectional, there are relatively small numbers of subjects (risking lack of statistical power to detect some associations), and there are no pre-earthquake measures of resilience. We cannot compare the 77 % responders to the 23 % non-responders. It would be difficult (and undesirable) to replicate the study. While the survey was undertaken 3-12 months after the major earthquakes, it is possible that other life events might also have contributed to individual students’ levels of stress. Ironically the 10,000 aftershocks were ‘helpful’ as it mitigates the potential problem of recall bias when trying to determine how the earthquakes affected a respondent. Furthermore, the PTSD questions were phrased deliberately in relation to the earthquake or aftershock in the previous 2 months that was the worst for the respondent. The strengths are the opportunity to evaluate the effect of a universal stressful event on a student population, the high response rates, the administration of the questionnaire after the stressful event but before the results of the exams are known to students, and the opportunity to remove the problem of determining reverse causality where poor performance may be the cause of the stress, not its effect. Finally, we did not look at the effect on examination performance raw scores – we looked at deviation from a student’s expected level of performance on examinations - thus controlling for student ability.

These findings lead us to build on the conceptual framework outlined by others [8–10]. We have confirmed that academic performance is assisted by students having a secure physical and emotional base. We have provided greater understanding into the relationship between the nature of a stressful event and its effect. In general, if the stressor is related to the task, there can sometimes be a beneficial effect. In contrast, if the stressor is peripheral to the task, performance is more likely to be impaired [10]. Our findings contribute to these concepts as we have found, in contrast, that a stressor peripheral to the task may not impair performance. Instead, we have found subgroups of students who are more vulnerable. The negative association between performance and feelings of support from the university suggest that some students’ responses to stress could well be stimuli to study harder, and over-compensate. This is consistent with the suggestion by others that different students respond in different ways to stress and this can be moderated, for example, by resourcefulness or other coping mechanisms [2].

Put another way, and consistent with existing research [8–10], provided students feel secure and safe at home, with good support networks, they can cope with stress at work. This highlights that exam stress cannot be seen as the same as life stress – much of the literature has looked at exam stress and its effects on performance. It would seem that not all stress leads to underperformance [10]. Instead, it is plausible that some of the associations between work stress and underperformance could be due to reverse causality – underperformance contributing to stress.

We found no association with scores on the Connor-Davidson resilience scale, which might suggest that resilience is not important here. In contrast, we have found an association between some conditions that promote resilience and examination performance – particularly, social support [36], and activities that facilitate relationships among faculty and trainees [19]. Resilience, as a concept, includes notions that what a person can do can make a difference and/or that they can have control of a situation [36]. Our interpretation of this suggests that, regardless of a person’s resilience, that during a series of earthquakes it would be reasonable for the most resilient person to conclude that even they cannot control what is happening.

## Conclusion

The students who are most vulnerable are those with fewer social networks, and those who are recent immigrants. As we have previously found, the learning environment seems to be less critical [20]. If a medical school wishes to use markers to detect vulnerable students, the findings of our study suggest that looking for students who are recent arrivals in the country or who have weaker social networks or support in their home environment may be the place to start.

### Ethical approval

The University of Otago Ethics Committee approved the study.
